# Mechanisms of Mycotoxin-Induced Neurotoxicity through Oxidative Stress-Associated Pathways

**DOI:** 10.3390/ijms12085213

**Published:** 2011-08-15

**Authors:** Kunio Doi, Koji Uetsuka

**Affiliations:** 1 Nippon Institute for Biological Science, 9-2221-1, Shin-Machi, Ome, Tokyo 198-0024, Japan; 2 Graduate School of Agricultural and Life Sciences, The University of Tokyo, 1-1-1, Yayoi, Bunkyo, Tokyo 113-8657, Japan

**Keywords:** neurotoxicity, T-2 toxin, macrocyclic trichothecenes, fumonisin B_1_, ochratoxin A

## Abstract

Among many mycotoxins, T-2 toxin, macrocyclic trichothecenes, fumonisin B_1_ (FB_1_) and ochratochin A (OTA) are known to have the potential to induce neurotoxicity in rodent models. T-2 toxin induces neuronal cell apoptosis in the fetal and adult brain. Macrocyclic trichothecenes bring about neuronal cell apoptosis and inflammation in the olfactory epithelium and olfactory bulb. FB_1_ induces neuronal degeneration in the cerebral cortex, concurrent with disruption of *de novo* ceramide synthesis. OTA causes acute depletion of striatal dopamine and its metabolites, accompanying evidence of neuronal cell apoptosis in the substantia nigra, striatum and hippocampus. This paper reviews the mechanisms of neurotoxicity induced by these mycotoxins especially from the viewpoint of oxidative stress-associated pathways.

## 1. Introduction

Mycotoxins are fungal metabolites known to be harmful toward human and animal health. To date, disorders caused by mycotoxins have been reported in digestive, urinary, immune and reproduction systems [1], and many *in vivo* and *in vitro* studies have been performed in order to clarify the mechanisms of mycotoxin-induced toxicity in these systems. Recently, Surai *et al.* [2] described that, in many cases, membrane-active properties of various mycotoxins determine their toxicity and incorporation of mycotoxins into membrane structures causes various detrimental changes, resulting in alterations in second messenger systems through damaging membrane receptors. In addition, detrimental effects of mycotoxins on DNA and RNA and protein synthesis together with proapoptotic action further compromise important metabolic pathways and consequently changes in physiological functions including growth, development and reproduction occur. During the last decades, the importance of oxidative stress and lipid peroxidation in all these processes have been pointed out by many researchers [2,3].

Compared with the amount of research on digestive, urinary, immune and reproduction systems, there are few reports of the effects of mycotoxins on neuronal tissues. This paper reviews the mechanisms of neurotoxicity experimentally induced in rats and mice by T-2 toxin, macrocyclic trichothecenes, fumonisin B_1_ (FB_1_) and ochratoxin A (OTA) especially from the viewpoint of oxidative stress-associated pathways.

## 2. T-2 Toxin

T-2 toxin is a cytotoxic secondary fungal metabolite that belongs to thetrichothecene mycotoxin family. They are produced by various species of *Fusarium* (*F. sporotichioides*, *F. poae*, *F. equiseti*, and *F. acuminatum*), which can infect corn, wheat, barley and rice crops in the field or during storage [4,5]. T-2 toxin is conjectured to be a major factor in alimentary toxic aleukia in humans [6] and has been implicated in additional mycotoxicoses such as red mold disease in humans and animals [7] and beanhull poisoning in horses [8].

T-2 toxin is a well-known inhibitor of protein synthesis through its high binding affinity to peptidyl transferase which is an integral part of the 60 s ribosomal subunit [9–11]. Subsequent inhibition of the peptidyl transferase reaction can trigger a ribotoxic stress response that activates c-Jun N-terminal kinase (JNK)/p38 mitogen-activated protein kinases (MAPKs) [11]. Moreover, T-2 toxin interferes with the metabolism of membrane phospholipids and increases liver lipid peroxides [12,13].

Oral, parenteral and cutaneous exposures to T-2 toxin induce lesions in hematopoietic, lymphoid and gastrointestinal tissues and suppress reproductive functions in domestic and laboratory animals [14–17]. T-2 toxin can induce apoptosis in many types of cells bearing rapid rates of proliferation [18–22]. T-2 toxin also induces apoptosis and fatty change in hepatocytes of mice following the increased expression of both oxidative stress- and apoptosis-related genes (c-fos and c-jun) [23]. Moreover, prenatal exposure of rats to T-2 toxin induces apoptosis in maternal liver, placenta and fetal liver following the increased expression of oxidative stress- and apoptosis-related genes and decreased expression of lipid metabolism- and drug-metabolizing enzyme-related genes in these tissues [24]. Doi *et al.* [25,26] have reviewed the mode of occurrence and mechanisms of T-2 toxin-induced apoptosis in mice and rats.

To date, the effects of T-2 toxin on the central nervous system (CNS) have received limited attention [27], and therefore, there are only a small number of reports of T-2 toxin-induced neurotoxicity [28–30]. Boyd *et al.* [28] reported that low levels of T-2 toxin were responsible for the changes in the metabolism of brain biogenic monoamines, and Wang *et al.* [31] showed that ingestion of T-2 toxin leads to changes in amino acid permeability across the blood-brain barrier, which could lead to neurological effects observed in animals exposed to trichothecenes. T-2 toxin may be easily distributed to the fetal brain, and induce fetal death and fetotoxicity mainly in the CNS and skeletal system in addition to maternal toxicity [32–35].

Sehata *et al.* [36] have investigated the mechanisms of apoptosis induction in the fetal brain by oral administration of T-2 toxin (2 mg/kg b.w.) to pregnant rats on day 13 of gestation. In their study, the number of apoptotic neural progenitor cells in the telencephalon increased from 1 h and peaked at 12 h after T-2 toxin treatment. Microarray analysis revealed that the expression of heat shock protein 70 (HSP70), metallothionein (MT)-2 and 1, and heme oxygenase-1 (HO-1) was strongly elevated by T-2 toxin at 12 h, and the expression of the Cu, Zn-superoxide dismutase (Cu, Zn-SOD) gene also increased at 24 h after T-2 toxin treatment. This suggests that oxidative stress might be the main factor behind the T-2 toxin-induced changes in the fetal brain. In addition, the gene expression of liver stearyl-CoA desaturase and farnesyl diphosphate synthase genes which are involved in lipid metabolism was suppressed by T-2 toxin in the fetal brain [36].

T-2 toxin suppresses drug metabolizing enzymes such as glutathione S-transferases (GSTs) [36–38]. In addition, a decreased expression in mitochondria-related genes, such as mitochondrial NADH-dehydrogenase and cytochrome oxidase, has been reported in the fetal brain [36], suggesting a dysfunction of the mitochondria. Since mitochondria play an important role in cell survival, these changes in metabolism-related genes may also have a relationship to the induction of apoptosis.

In the study by Sehata *et al.* [36] on the fetal brain, the expression of MEKK1 gene increased at 12 and 24 h, and the expression of c-jun gene at 24 h after T-2 toxin treatment. These findings suggest that the MAPK-JNK-c-jun pathway might be involved in T-2 toxin-induced apoptosis in the fetal brain. Extracellular signal-related protein kinase (ERK) mediates cell growth and protects cells from apoptosis, whereas stress-activated protein kinase (SAPK)/JNK and p38 MAPK inhibit cell proliferation and may promote apoptosis [39]. Each MAPK is activated by an upstream MAPK kinase, including MEKK1, and JNK activates transcription factors such as c-fos and c-jun. MEKK1 may induce apoptosis by causing a general deregulation of MAPK signaling [40], and JNK and c-jun are important regulators of apoptosis in the nervous system [41].

Differing from the results in the maternal liver, placenta and fetal liver [24], the increase in caspase-2 gene expression with no changes in caspase-9 and Bax-α gene expression was detected in the fetal brain at 24 h after T-2 toxin treatment [36], suggesting an involvement of caspase-2 activation in T-2 toxin-induced apoptosis in the fetal brain. Activation of caspase-2 is induced by reactive oxygen species (ROS), and caspase-2 is said to play a crucial role in the control of apoptosis [42–44]. Although it is suggested that the p53-related mitochondrial pathway is involved in the T-2 toxin-induced apoptosis in the maternal and fetal livers [24], apoptosis induction in the fetal brain by T-2 toxin seems to be independent of the p53-related pathway which is the most important pathway in DNA-damaging agent-induced apoptosis of neural progenitor cells in the developing brain [45–49].

In microarray analysis on the fetal rat brain from dams exposed to T-2 toxin, the expression of vascular endothelial growth factor (VEGF) gene increased at 12 and 24 h after T-2 toxin treatment [36]. VEGF is expressed in neurons and may play a role in the maintenance of neurons and endothelial cells in the CNS [50]. Therefore, the observed VEGF induction in the fetal brain might indicate a protective reaction to the apoptotic changes in the fetal brain induced by T-2 toxin.

Recently, Chaudhary and Rao [27] evaluated acute toxicity of dermal and subcutaneous exposure of T-2 toxin on brain oxidative stress in adult mice. Mice were exposed to LD_50_ of T-2 toxin either by the dermal (5.94 mg/kg b.w.) or subcutaneous (1.54 mg/kg b.w.) route and sacrificed at 1, 3 and 7 days post-exposure. They reported that T-2 toxin-treated animals showed a time-dependent increase in ROS generation, glutathione (GSH) depletion, lipid peroxidation and protein carbonyl content in the brain in both routes of exposure. The gene expression profile of antioxidant enzymes showed a significant increase in SOD and catalase via the percutaneous route and glutathione reductase (GR) and glutathione peroxidase (GPx) via the subcutaneous route. This indicates that T-2 toxin induces oxidative damage in adult mouse brain as well as in fetal rat brain. Lipid peroxidation may bring about protein damage and inactivation of membrane-bound enzyme either through direct attack by free radicals or through chemical modification by its end products [51]. Indeed, as mentioned above, protein carbonylation, a sign of oxidative damage, significantly increased in the mouse brain after exposure to T-2 toxin.

Chaudhary and Roa [27] also investigated the role of nuclear factor erythroid 2-related factor (Nrf2) and its downstream targets of phase II antioxidant/detoxifying enzymes in the mouse brain exposed to T-2 toxin. Upon activation, Nrf2 binds to antioxidant responsive element sites in the promoter regions of many detoxification and antioxidant genes, leading to coordinate regulation of downstream targets that boost the cellular detoxification process and antioxidant potential [52,53]. In the study of Chaudhary and Roa [27], however, Nrf2 and its downstream target genes were down-regulated, and the involvement of Nrf2 in augmenting oxidative potential is not significant, although there is oxidative stress.

## 3. Macrocyclic Trichothecenes

The fungus *Stachybotrys chartarum*, a saprophyte that grows on wet cellulose-containing building materials including wallboard, ceiling tiles and cardboard is often found in low concentrations among the mycoflora identified in water-damaged buildings [54–57]. Chronic indoor exposures to *S. chartarum* and its products or components have been postulated to etiologically contribute to damp building-related illnesses (DBRI) such as debilitating respiratory [58,59] and nonrespiratory symptoms involving immune and neurological impairment [56,60–62]. Experimental rodent studies revealed that, while this fungus is not infectious, airway exposure to spores of *S. chartarum* and its components have the potential to evoke toxicity, inflammation and allergic sensitization in the upper and lower respiratory tracts [56].

Two toxic “chemotypes” of *S. chartarum* exist. One chemotype elaborates highly toxic macrocyclic trichotecene mycotoxins whereas a second chemotype produces less toxic atranones and simple trichothecenes but no macrocyclic trichothecenes [63]. The former mycotoxins are potent translational inhibitors and stress kinase activators that appear to be a critical underlying cause for a number of adverse effects. Notably, these toxins form covalent protein adducts *in vitro* and *in vivo* and, furthermore, cause neurotoxicity and inflammation in the nose and brain of the mouse [64,65]. On the other hand, the latter mycotoxins can induce pulmonary inflammation. Pestka *et al.* [56] have reviewed the relationship between *S. chartarum*, trichothecene mycotoxins, and DBRI and proposed new insights into a public health enigma.

Besides inhibiting translation, macrocyclic trichothecenes as well as other trichothecenes can simultaneously activate p38, JNK and ERK and MAPKs *in vivo* and *in vitro* [66–68] via a process referred to as “ribotoxic stress” [69]. In cell cultures, macrocyclic trichothecenes (Type D trichotecenes) are 10–100 times more potent than Type A (e.g., T-2 toxin) or Type B (e.g., deoxynivalenol) trichothecenes at activating MAPKs, impairing leukocyte proliferation, or inducing apoptosis [11,70–75]. The common ability of macrocyclic trichothecenes to cause protein synthesis inhibition via binding to the 18S rRNA of the ribosomal large subunit [76] has been speculated to be a major mechanism underlying induction of cell apoptosis by this group of trichothecenes. Moreover, the potential of macrocyclic trichothecenes to covalently bind to proteins and possibly other macromolecules has major implications relative to their absorption, metabolism, distribution, toxicity, and potential allergenicity [64,65].

Satratoxin G (SG) is one of the most potent macrocyclic trichothecenes produced by *S. chartarum* [75] and contributes to the above-mentioned DBRI. Roridin A (RA) is a commercially available macrocyclic trichithecene used as a SG surrogate, and roridin L2 (RL2) is a putative biosynthetic precursor of SG. While SG contains an intact macrocyclic ring linking C-4 to C-15, the precursor RL2 contains only an extended carbon chain linked at C-4 [77]. RL2 is said to be nontoxic [78]. Satratoxin H (SH) is another macrocyclic trichothecene mycotoxin derived from the fungus *S. chartaum*. This mycotoxin is one of the toxic constituents of the toxic mushroom, *Podostoma cornu-damae* [79].

Murine alveolar type II cells and alveolar macrophages are extremely sensitive to intratracheally instilled *S. chartarum* spores [80]. The methanol extract of a trichothecene-producing strain of *S. chartarum* particularly up-regulates DNA damage-responsive and DNA repair genes in the murine alveolar macrophage cell line MH-S early in the treatment, which are suggestive of genotoxic stress [81]. In a follow-up study, extract-induced apoptosis in MH-S cells was observed to precede DNA damage [82]. Moreover, both p38- and p53-mediated signaling events seem to occur in *S. chartarum*-induced apoptosis of alveolar macrophages.

Macrocyclic tricothecenes also affect the upper respiratory tract (e.g., nasal airway). Using an intranasal instillation model in adult C57BL/6J mice, Islam *et al.* [83] showed that SG exposure specifically induced apoptosis of the olfactory sensory neurons (OSNs) and subsequent atrophy of the olfactory epithelium (OE). Concurrently, there was bilateral atrophy of the olfactory nerve layer of the olfactory bulbs (OBs) of the brain. In addition, SG induced an acute, neutrophilic rhinitis and encephalitis. Similar findings have also been reported in mice intranasally instilled with RA [84]. In the ethmoid turbinates and OBs in the frontal brain in mice treated with SG, elevated mRNA expression for the proinflammatory cytokines, TNF-α, IL-6 and IL-1, the chemokine macrophage-inflammatory protein-2 (MIP-2), and the proapoptotic genes, Fas, FasL, p75NGFR, p53, Bax, caspase-3 and CAD, was detected at 24 h post instillation (PI). In the same regions of mice treated with RA, up-regulated mRNA expression of Fas, TNF-α, IL-6 and IL-1 and MIP-2 was observed from 6 to 24 h PI, whereas expression of several other proapoptotic genes (p53, Bax, and caspase-activated DNase) was detectable only at 24 h PI.

Following intranasal instillation of mice to SG [83] or RA [84], double-stranded RNA-activated protein kinase (PKR) mRNA concentrations in the nasal turbinates were up-regulated in parallel with OSN apoptosis. PKR, Bax and p53 have been previously reported to mediate apoptosis in murine OSNs [85–87]. PKR associates with the ribosome [88] and can selectively shut down translation via phosphorylation of eukaryolic initiation factor 2α (eIF2α) as well as activate NF-κB [89].

Both TNF-α and Fas directly induce apoptosis in OE [90] and in OE organ cultures [91,92], and SG- and RA-induced TNF-α and Fas mRNA expression precedes or is concurrent with OSN apoptosis, induction of caspase-3 mRNA and caspase-3 activation [83,84]. The origins of induced TNF-α are considered to be OSNs and adjacent cells in the OE which would promote autocrine or paracrine responses, respectively. SG and RA also directly induces apoptosis in the OSNs by initiating mitochondrial cell death via an intrinsic pathway involving p53 and Bax. Islam *et al.* [93] used the PC12 rat pheochromocytoma cell models to elucidate potential mechanisms of SG-induced neuronal cell death. In their experiment, SG-induced apoptosis occurred at 48 h after SG treatment, and the expression of p53, and PKR, Bax and caspase-activated DNase mRNAs was significantly elevated from 6 to 48 h after SG treatment. SG-induced p53 and Bax gene expression is known to drive nuclear translocation of apoptosis-inducing factor (AIF), mitochondrial flavoprotein, in PC-12 cells [93].

In the study by Islam *et al*. [93], SG-induced apoptosis was not affected by inhibitors of oxidative stress or MAPKs but was suppressed by the PKR inhibitor C16 and by PKR siRNA transfection. PKR inhibition also blocked SG-induced apoptotic gene expression and AIF translocation but not caspase-3 activation. These results indicate that SG-induced apoptosis in PC12 neural cells is mediated by PKR via a caspase-independent pathway possibly involving AIF translocation.

SH is thought to induce caspase-3 activation and apoptosis of PC12 cells through the activation of p38 MAPK and JNK in a GSH-sensitive manner [94]. Moreover, Nusuetrong *et al.* [79] carried out the study to further elucidate the mechanisms by which SH induces cell death in PC12 cells. They reported that SH causes apoptosis of serum-deprived PC12 cells within 24 h and that SH increases ROS production and lipid peroxidation which are attenuated by incubation of cells with GSH. They suggested that SH-induced increase in apoptosis of serum-deprived PC12 cells, may be partially mediated through the generation of ROS. GSH, the most abundant intracellular thiol, plays an important role in controlling the redox state of cells, and GSH is thought to play a role in apoptotic cell death following its efflux through the GSH-specific membrane channels, carrier-mediated GSH extrusion and oxidative stress [79,95].

The constant activation of inflammatory and apoptotic pathways at low levels of exposure in human neurological system cells may amplify devastation to neurological tissues and lead to neurological system cell damage from indirect events triggered by the presence of SH [96]. This suggests that individuals exposed to SH and microbial organisms resulting in a chronic immune response (inflammation and oxidative stress) could have increased sensitivity to these agents, leading to neural damage, further supporting previous *in vivo* studies demonstrating CNS tissue damage via inhalation of fungal toxins [97]. The process of inflammation is intended to repair injured tissues; however, this mechanism tends to induce damage to nervous tissues when activated [98,99]. In this context, studies on canines by Caldeón-Garcidueñas *et al.* [100,101] demonstrated increased iNOS, NF-κB, and TNF-α production among other inflammatory and oxidative stress agents—leading to permanent damage of DNA and CNS tissues due to passage of small particles via olfactory epithelium and lung tissue. The fine particles reach CNS tissue via the olfactory bulb and into brain tissue (frontal cortex and cortical tissues) demonstrating increases in β-amyloid plaques suggestive of pathogenesis similar to Alzheimer’s disease [100,101].

## 4. Fumonisin B_1_

Fumonisins belong to the relatively recently discovered group of mycotoxins produced by the fungus *Fusarium verticillioides* (formerly *F. moniliforme*), a widespread fungal concomitant of various cereals, predominantly corn [2,102]. In this case, FB_1_ is the most abundant and toxic; it has been linked to a number of diseases in humans and animals [102,103].

The structures of FB_1_ and sphingolipids show marked similarities, which may be the reason why FB_1_ drastically disrupts the normal sphingolipid metabolism [104]. FB_1_ inhibits ceramide synthase, a key enzyme in *de novo* sphingolipid biosynthesis and sphingolipid turnover, causing elevated levels of free sphingolipid bases and sphingolipid base metabolites and lowered levels of ceramide [105,106]. FB_1_-induced inhibition of ceramide synthesis can result in a wide spectrum of changes in lipid metabolism and associated lipid-depending signaling pathways, and it appears to be a major contributor to the carcinogenic and other deleterious effects of FB_1_ [106].

FB_1_ is well known to cause equine leukoencephalomalacia (ELEM) [107,108]. This disease is associated primarily with FB_1_ and is characterized by high mortality [108]. In histopathological examinations, pathognomonic focal necrotic lesions, located primarily in the subcortical white matter are apparent [102]. In addition, the elevation of free sphingoid bases after FB_1_ treatment has been demonstrated in the brain of ELEM-diagnosed horses [109], suggesting that free sphingoid bases may be important in FB_1_-related neurotoxicity.

Another emerging neurodevelopmental aspect of FB_1_ toxicity that implicates the consumption of fumonisins in the etiology of neuronal tubule defects (NTD) in children has recently been suggested [110]. Treatment with FB_1_ causes NTDs in *ex vivo* neurulating mouse embryos [111] and this effect is related to the folic acid receptor deficiency as a result of the FB_1_-dependent lipid rafts depletion [112].

The detrimental effects of FB_1_ on neuronal tissue have been shown in a number of reports indicating its potential for direct neurotoxicity. For example, FB_1_ drastically inhibits axonal growth in cultured hippocampal neurons [113], increases levels of sphinganine concentration in the forebrain and brain stem of rats accompanying a concomitant demyelination in the forebrain [114], and disrupts myelination in glial cells but not neurons in aggregating brain cell culture [115] and in developing rats [116]. FB_1_-dependent changes in neurotransmitter metabolite levels in different brain regions of BALB/c mice [117] and in rat brain [118], and alteration of electrophysiological activity in rat neocortex [199] are also reported.

Osuchowski *et al.* [120] carried out a study to compare the toxicity of FB_1_ in mouse brain after an intracerebroventricular (icv) or subcutaneous (sc) infusion with total doses of 0, 10 or 100 μg/kg of FB_1_. The icv infusion of FB_1_ led to neuronal degeneration in the cortex, concurrent with disruption of sphingolipid metabolism, i.e., inhibition of *de novo* ceramide synthesis, stimulation of astrocytes, and activation of proinflammatory cytokine signaling while the sc infusion of FB_1_ brought about partial inhibition of sphingolipid metabolism in the cortex. From these results and the reports showing that FB_1_ compromises the endothelial barrier function [121,122], it is suggested that there may be limited blood-brain barrier transfer of FB_1_ [123] and that FB_1_ may disrupt central nervous system homeostasis when brain tissue is directly exposed to this mycotoxin.

All cytokines analyzed in the study of Osuchowski *et al.* [120] are CNS-borne and are expressed on-site by neurons (IL-1β and IL-6), astrocytes (IL-1β, IL-6, TNF-α and interferon-γ; IFN-γ) and microglia (IL-1β, IL-6 and TNF-α) [124]. IL-1β, IL-6 and TNF-α are primarily associated with neuronal injury; thus neuronal damage will be accompanied by their elevated expression. In addition, the increased expression of TNF-α and IL-1β also indirectly enhance neuronal damage via ceramide-mediated signaling, since both cytokines activate brain neutral sphingomyelinase (nSMase), and the role of ceramide-dependent neurodegeneration mediated by nSMase is reported [125,126]. The immunocompetent cells and proinflammatory signaling are first being activated (i.e., astrocytes and probably microglia) and then neurodegeneration follows in mouse brain after FB_1_ infusion [120].

During the last decades, studies aimed at clarifying the mechanisms of FB_1_-induced neurotoxicity in cultured cells has been done mainly from the viewpoints of oxidative stress and/or apoptosis. Stockmann-Juvala *et al.* [127] tried to characterize oxidative stress-related parameters induced by FB_1_ in three different neural cell lines, human SH-SY5Y neuroblastoma, rat C6 glioblastoma and mouse GT1-7 hypothalamic cells. In their study, FB_1_ caused a dose-dependent increase of ROS production in C6 and GT1-7 cells but was without an effect in SH-SY5Y cells. Decreased GSH levels, increased malon dialdehyde (MDA)-formation, indicative of lipid peroxidation, and necrotic cell death were observed in all cell lines after incubation with FB_1_. From these results, they concluded that FB_1_ induces oxidative stress in human, rat and mouse neural cell cultures. They also suggested that FB_1_ is cytotoxic to neural cells only at high concentrations *in vitro*, although systemic toxicity, which may be caused by the inhibition of ceramide synthase, takes place already at very low concentrations of FB_1_ [104].

Mobio *et al.* [128–130] reported that in rat C6 glioma cells, FB_1_ inhibits protein synthesis, causes DNA fragmentation and cell death, increases 8-hydroxy-2′-deoxyguanosine (8-OH-dG), and induces lipid peroxidation, and that cytotoxic concentrations of FB_1_ induce cell cycle arrest in C6 cells (in the G2/M phase after 24 h and in G0/G1 after 48 h incubation with FB_1_), possibly associated with genotoxic event. On the other hand, Galvano *et al.* [131,132] reported that FB_1_ does not increase ROS production or cell death in rat astrocytes although DNA-damage and caspase-3 activation take place. Based on these results, the authors suggested that effects of FB_1_ are not a result of oxidative injury, but are instead a response that may occur after modulation of protective genes. These theories support the above-mentioned observations in FB_1_-treated SH-SY5Y cells. In SH-SY5Y cells, lipid peroxidation took place without an increase in ROS production, and was associated with delayed cell death. Moreover, low expression of the anti-apoptotic Bcl-2 protein in GT1-7 and C6 cells can be linked to low basal GSH levels in these cell lines [127], and may increase their susceptibility to radical attack [133–135]. SH-SY5Y cells, on the other hand, express higher levels of Bcl-2 [133,136], which may explain why GSH levels in these cells decreased later than in GT1-7 and C6 cells exposed to FB_1_.

Stockmann-Juvala *et al.* [137] have also investigated the effects of FB_1_ on human U-118MG glioblastoma cells. In their study, FB_1_ increased lipid peroxidation and the production of ROS in U-118 MG cells dose- and time-dependently and these effects were accompanied by decreases in the GSH levels and cell viability. In addition, signs of apoptosis were indicated by increased caspase-3-like protease activity and internucleosomal DNA fragmentation. Based on these results, they concluded that oxidative stress and apoptosis may be involved in the neurotoxicity induced by FB_1_. There are a number of studies showing FB_1_-induced apoptosis in different cell types [129–131,138–140]. Apoptosis is considered to be a common result of oxidative stress caused by ROS production, disturbance of GSH generation and lipid peroxidation [141,142]. In addition, activation of caspase-3 may be one of the events causing an increase in ROS production, and subsequent lipid peroxidation and reduction of intracellular GSH levels.

## 5. Ochratoxin A

Ochratoxin A (OTA) is a fungal metabolite produced by *Aspergillus ochraceus* and *Penicillium verrucosum*. OTA is found in a variety of plant food products such as cereals. Because of its long half-life, it accumulates in the food chain [143,144] and is frequently detected in human plasma at nanomolar concentrations [145,146]. OTA has been found to be involved in the developments of certain kidney diseases [147] and enzymuria [148] similar to Balkan endemic nephropathy found in humans [147,149]. In addition to nephrotoxicity, the main OTA-induced toxicity, OTA has also been reported to have immunotoxic [150,151], teratogenic [152,153], genotoxic [154] and neurotoxic [155] effects. Although there is still insufficient evidence in humans, there is sufficient evidence in experimental animals for the carcinogenicity of OTA [156].

OTA has complex mechanisms of action that include evocation of oxidative stress, bio-energetic compromise, mitochondrial impairment, inhibition of protein synthesis, production of DNA single-strand breaks and formation of OTA-DNA adducts [155,157–160]. The bio-energetic compromise induced by OTA may be responsible for the generation of free radicals and ROS that results in global oxidative damage to DNA and lipids and damage to proteins through generation of oxygen free radicals and nitric oxide [161,162]. OTA renal toxicity and carcinogenicity may be, at least partly, mediated by an Nrf2-dependent signal transduction pathway [53]. Effect of OTA on potential signaling molecules (growth factors, fatty acids, and/or Ca2^+^) could disrupt PKC-regulated pathways downstream, and down-regulation of genes under transcriptional control of Nrf2 may lead to a reduced oxidative stress response. In addition, OTA-induced down-regulation of genes under hepatocyte nuclear factor 4α(HNF4α)-control may affect key metabolic processes. This could make kidney cells more vulnerable to OTA-induced toxicity leading to tumor development [163]. OTA has also been reported to inhibit succinate-dependent electron transfer in the electron transport chain, but at higher concentrations will also inhibit electron transport at Complex I [164,165], suggesting mitochondrial toxicity.

The developing brain appears to be very susceptible to the deleterious effects of OTA [166–169]. OTA has been shown to affect proliferation and migration of neurons [153] and reduce DNA content [170] in the developing rodent brain. OTA is also reported to be neurotoxic to adult male rats [166]. Neurotoxicity is more pronounced in the ventral mesencephalon, hippocampus (HP), and striatum than in the cerebellum (CB) [171]. Recent animal and cellular studies have suggested that OTA may contribute to the development of human and animal systemic problems, including neurodegenerative diseases and brain dysfunction [155].

Sava *et al.* [155] investigated the time course of acute effects of OTA in the context of DNA damage, DNA repair and global oxidative stress across six brain regions, CB, cortex (CX), HP, midbrain (MB), caudate/putamn (CP) and pons/medulla (PM), in male mice, and they showed that OTA causes acute depletion of striatal dopamine (DA) and its metabolites as well as decreased tyrosine hydroxylase immunoreactivity in the corpus striatum on a background of globally increased oxidative stress evidenced by significant increases in lipid peroxidation and oxidative DNA damage, and transient inhibition of oxidative DNA repair activity (oxyguanosine glyosylase, OGG1) across six brain regions, accompanying evidence for apoptosis in the substantia nigra (SN), striatum and HP or other regions. Unlike the monophasic SOD activation, the oxidative DNA repair response exhibited a biphasic response.

Sava *et al.* [172] also examined the possibility that OTA can cause parkinsonism in male mice focusing on the effects of chronic low doses of OTA exposure on regional brain oxidative stress and striatal DA metabolism. They reported that continuous administration of low doses of OTA with implanted subcutaneous Alzet minipumps caused a small but significant decrease in striatal DA levels and an up-regulation of anti-oxidant systems and DNA repair. These data suggest the possibility that low dose exposure to OTA will result in an earlier onset of parkinsonism when normal age-dependent decline in striatal DA levels is superimposed on the mycotoxin-induced lesion. In their study, since the CP and MB showed a relatively diminished OGG1 activity and increased oxidative DNA damage, it was postulated that the DA terminals of the striatum would suffer damage. This concept is supported by an earlier report of increased oxidative DNA damage in SN and striatum in post-mortem brain from Parkinson’s disease cases [173] and by the above-mentioned report that acute doses of OTA caused a dose-dependent decrease of striatal DA and a decrease in DA turnover [155]. The regional vulnerability to the toxin was not directly related to the concentration of the toxin in each region. Moreover, as mentioned above, not all regions were equally sensitive to the toxin, even though all brain regions were capable of marked increases in OGG1 activity. Namely, the CP was most sensitive to the toxin while the CB was the least sensitive. In addition, the HP, a primary site of neurodegeneration in Alzheimer’s disease, turns out to exhibit relatively high OTA levels with concurrently pronounced OTA neurotxicity [166]. OTA may also be toxic through other mechanisms. For example, due to its chemical structure, OTA inhibits protein synthesis by competition with phenylalanine in the aminoacylation reaction of phenylalanine-tRNA [174,175] and phenylalanine hydroxylase activity [157], leading to the impairment of the synthesis of DOPA, dopamine and catecholamines or enzymes involved in the metabolism of DNA.

The adult brain retains a reservoir of stem-progenitor cells in the hippocampal “neurogenic zone” capable of proliferative activity throughout life [176,177], and it is known that injury, irradiation, drugs and endogenous factors such as hormones and trophic factors impact neurogenesis [178–181]. Therefore, OTA may also impact neurogenesis in adult HP. In addition, subchronic administration of OTA is demonstrated to affect cognitive functions by reducing hippocampal *N*-methyl-d-aspartate (NMDA) receptor subunits 2A and 2B concentrations in rats [182].

Sava *et al.* [183] tested neural stem/progenitor cells (NSCs) prepared from HP of adult mouse brain for their vulnerability to OTA *in vitro*. OTA, added to the cultures in concentrations of 0.01–100 mg/mL, caused a dose- and time-dependent (6–72 h) decrease in viability of both proliferating and differentiating NSC. Along with decreased viability, OTA elicited a pronounced oxidative stress evidenced by a robust increase in total and mitochondrial SOD activity, and OTA also significantly increased OGG1 activity. Proliferating NSC exhibited a greater vulnerability to the toxin than differentiated neurons despite robust DNA repair and antioxidant responses. Such a result is unexpected since DNA repair systems are typically more active and efficient in proliferating cells than in post-mitotic differentiated cells [184,185]. It suggests that OTA’s mechanism of action as an inhibitor of mitochondrial oxidative metabolism may be less critical than its interference with DNA synthesis and mitotic competence. Overall, Sava *et al.* [183] speculated that OTA exposure may contribute to impaired hippocampal neurogenesis *in vivo*, resulting in depression and cognitive deficits, conditions reported to be linked to mycotoxin exposure in humans [186–188].

Yoon *et al.* [189] investigated the potential harm caused by environmental exposure to OTA in terms of its effects on neuronal cell viability and proteome profiles using mouse hippocampal HT22 and human neuroblastoma SH-SY5Y cells. Generation of ROS was detected in OTA-treated SH-SY5Y and HT22 cells, however, caspase activation and an increase in p53 phosphorylation were only detected in HT22 cells, even though OTA treatment caused oxidative stress in both two cell lines. The expressions of several proteins (valosin containing protein, propyl 4-hydroxylase, Atp5b protein, nucleophosmin 1, eukaryotic translation elongation factor 1 delta isoform, ornithine aminotransferase, prohibitin, and peroxiredoxin 6), which have been suggested to be implicated in the pathogenesis of neurodegenerative disorders, were up-regulated only in HT22 cells after treatment with OTA, which was interesting because OTA induced the apoptosis of HT22 cells but not of SH-SY5Y cells. Involvement of the mitochondrial dysfunction-related apoptotic process in OTA toxicity in HT22 cells was corroborated by the results showing significant decline in mitochondrial activity in OTA-treated HT22 cells, not in SH-SY5Y cells. Such differences between cell lines might be due in part to the complex natures of protein expression and functional regulation required during the intracellular signaling of apoptosis [190], and the above-mentioned alteration of protein expression profile in HT22 cells after OTA treatment is considered to be related to ROS generation. Because inhibition of expression of propyl 4-hydoxylase is known to attenuate neuronal death associated with oxidative stress [191], ornithine has been shown to be up-regulated during ROS-related apoptotic cell death [192], and valosin containing protein has been proposed to contribute to the conversion of oxidative stress to an endoplasmic reticulum stress response during the pathological processes of a number of neurodegenerative disorders [193]. Contrary to the above-mentioned report by Sava *et al.* [183], Zhang *et al.* [194] reported that caspase-9 and caspase-3 were activated in response to OTA treatment and caspase inhibitors were effective in partly counteracting OTA-induced apoptosis-related neurocytotoxicity not only in primary rat cortical neuronal cells but also in human neuroblastoma SHSY5Y cells and that such OTA-induced apoptosis was accompanied by a loss of mitochondria membrane potential. The authors suggest that OTA may contribute to the pathogenesis of neurodegenerative diseases (e.g., Alzheimer’s and Parkinson’s disease) in which apoptotic processes are centrally involved. The reason for the difference in apoptosis-inducing ability of OTA in human neuroblastoma SH-SY5Y cells between the two reports still remains unclear.

Besides the toxic effects of OTA on neuronal cells, Zurich *et al.* [195] investigated the relationship between OTA toxicity and glial reactivity in serum-free aggregating rat brain cell cultures, and they showed that OTA affects the cytoskeletal integrity of astrocytes as well as the expression of genes pertaining to the brain inflammatory response system, and suggested that a relationship exists between the inflammatory events and the cytoskeletal changes induced by OTA. Furthermore, they also suggested that, by inducing an atypical glial reactivity, OTA may severely affect the neuroprotective capacity of glial cells. Moreover, Hong *et al.* [196] reported that OTA caused concentration-dependent reductions in neurite outgrowth and cell number, and induced the activation of transcription factors activator protein-1 (AP-1) and NF-κB activation in cultured rat embryonic midbrain cells, and that 15-deoxy-delta 12, 14-prostaglandin J2 (15-deoxy PGJ2), a peroxisome proliferator-activated receptor gamma (PPAR-γ) agonist, blocked OTA-induced neurotoxicity by inhibiting AP and NF-κB activation in cultured rat embryonic midbrain cells.

## 6. Conclusions

This paper has reviewed the mechanisms of neurotoxicity induced in rodents and neuronal cell lines by T-2 toxin, macrocyclic trichothecenes, FB_1_ and OTA.

T-2 toxin, one of the Type A trichothecene mycotoxins, triggers a ribotoxic response through its high binding affinity to peptidyl transferase which is an integral part of the 60 s ribosomal subunit, resulting in activation of JNK/p38 MAPKs. T-2 toxin also interferes with the metabolism of membrane phospholipids and increases liver lipid peroxides. In the fetal brain, oxidative stress is considered to be the main factor behind the T-2 toxin-induced changes, and the MAPK-JNK-c-jun pathway is thought to be involved in T-2 toxin-induced neuronal cell apoptosis. Moreover, activation of caspase-2 is essential to T-2 toxin-induced apoptosis in the fetal brain. T-2 toxin also induces oxidative damage in the adult mouse brain as well as in the fetal rat brain.

Macrocyclic trichothecenes have been postulated to etiologically contribute to DBRI such as debilitating respiratory and nonrespiratory symptoms. The common ability of macrocyclic trichothecenes to cause protein synthesis inhibition via binding to the 18s rRNA of the ribosomal large subunit has been speculated to be a major mechanism underlying induction of cell apoptosis by this group of trichothecenes. In mice, SG or RA exposure specifically induces apoptosis of OSNs and subsequent atrophy of OE. SG or RA also induces apoptosis in, and atrophy of, the olfactory nerve layer of OBs of the brain. Moreover, in the ethmoid turbinates and OBs in the frontal brain in mice treated with SG or RA, elevated mRNA expression for the inflammatory cytokines, chemokine, and proapoptotic genes and increased mRNA concentrations for PKR are detected. In PC12 neural cells, SG-induced apoptosis is mediated by PKR via a caspase-independent pathway possibly involving AIF translocation from mitochondria to the nucleus. On the other hand, SH is thought to induce caspase-3 activation and apoptosis of PC12 cells through the activation of MAPK and JNK in a GSH-sensitive manner.

FB_1_ is well known to cause ELEM and may be implicated in the etiology of NTD in children. FB_1_-induced inhibition of ceramide synthesis can result in a wide spectrum of changes in lipid metabolism and associated lipid-dependent pathways. FB_1_ may disrupt central nervous system homeostasis when brain tissue is directly exposed to this mycotoxin. Namely, the icv infusion of FB_1_ leads to neuronal degeneration in the cortex, concurrent with disruption of sphingolipid metabolism, i.e., inhibition of *de novo* ceramide synthesis, stimulation of astrocytes, and activation of proinflammatory cytokine signaling. In *in vitro* studies, FB_1_ inhibits protein synthesis, causes DNA fragmentation and cell death, increases 8-OH-dG, and induces lipid peroxidation in rat C6 glioma cells, and oxidative stress and apoptosis may be involved in the neurotoxicity induced in human U-118MG glioblastoma cells by FB_1_. On the other hand, effects of FB_1_ are not a result of oxidative injury but are instead a response that may occur after modulation of protective genes in rat astrocyte and SH-SY5Y human neuroblastoma cell cultures.

OTA has complex mechanisms of action that include evocation of oxidative stress, bio-energetic compromise, mitochondrial impairment, inhibition of protein synthesis, production of DNA single-strand breaks and formation of OTA-DNA adducts. OTA causes acute depletion of striatal DA and its metabolites in the corpus striatum on a background of globally increased oxidative stress across six brain regions of rats examined, accompanying evidence for apoptosis in the SN, striatum and HP or other regions. The CP is most sensitive to the toxin while the CB is the least sensitive, and the HP, primary site of neurodegeneration in Alzheimer’s disease, turns out to exhibit relatively high OTA levels with concurrently pronounced OTA neurotoxicity. OTA exposure may contribute to impaired hippocampal neurogenesis *in vivo*, resulting in depression and cognitive deficits. OTA also induces oxidative stress and apoptosis through caspase activation and increase in p53 phosphorylation in various neural cell cultures. OTA seems to contribute to the pathogenesis of neurodegenerative diseases in humans (e.g., Alzheimer’s and Parkinson’s disease), in which apoptotic processes are essentially involved.
